# Translation of ERC resuscitation guidelines into clinical practice by emergency physicians

**DOI:** 10.1186/1757-7241-22-9

**Published:** 2014-01-30

**Authors:** Henrik Fischer, Kaspar Bachmann, Guido Strunk, Stephanie Neuhold, Bernhard Zapletal, Claudia Maurer, Andrea Fast, Dominik Stumpf, Robert Greif

**Affiliations:** 1 Department of Anaesthesia, General Intensive Care and Pain Control, Division of Cardiothoracic and Vascular Anaesthesia and Intensive Care, Medical University Vienna, Währinger Gürtel 18-20, 1090 Vienna, Austria; 2 Department of Anaesthesiology and Pain Therapy, University Hospital Bern and University of Bern, Bern, Switzerland; 3 University of Technology, Dortmund, Germany; 4 Complexity Research, Vienna, Austria; 5 Department of Anaesthesia, General Intensive Care and Pain Control, Medical University Vienna, Vienna, Austria; 6 Hanusch Hospital, Vienna, Austria; 7 Landesklinikum Mödling, Mödling, Austria; 8 Hospital of the Sisters of Charity Linz, Linz, Austria

**Keywords:** Advanced life support, Emergency physicians out-of-hospital, Mild hypothermia, Intraosseous access, Clinical practice

## Abstract

**Purpose:**

Austrian out-of-hospital emergency physicians (OOHEP) undergo mandatory biannual emergency physician refresher courses to maintain their licence. The purpose of this study was to compare different reported emergency skills and knowledge, recommended by the European Resuscitation Council (ERC) guidelines, between OOHEP who work regularly at an out-of-hospital emergency service and those who do not currently work as OOHEP but are licenced.

**Methods:**

We obtained data from 854 participants from 19 refresher courses. Demographics, questions about their practice and multiple-choice questions about ALS-knowledge were answered and analysed. We particularly explored the application of therapeutic hypothermia, intraosseous access, pocket guide use and knowledge about the participants’ defibrillator in use. A multivariate logistic regression analysed differences between both groups of OOHEP. Age, gender, years of clinical experience, ERC-ALS provider course attendance and the self-reported number of resuscitations were control variables.

**Results:**

Licenced OOHEP who are currently employed in emergency service are significantly more likely to initiate intraosseous access (OR = 4.013, p < 0.01), they initiate mild-therapeutic hypothermia after successful resuscitation (OR = 2.550, p < 0.01) more often, and knowledge about the used defibrillator was higher (OR = 2.292, p < 0.01). No difference was found for the use of pocket guides.

OOHEP who have attended an ERC-ALS provider course since 2005 have initiated more mild therapeutic hypothermia after successful resuscitation (OR = 1.670, p <0.05) as well as participants who resuscitated within the last year (OR = 2.324, p < 0.01), while older OOHEP initiated mild therapeutic hypothermia less often, measured per year of age (OR = 0.913, p <0.01).

**Conclusion:**

Licenced and employed OOHEP implement ERC guidelines better into clinical practice, but more training on life-saving rescue techniques needs to be done to improve knowledge and to raise these rates of application.

## Introduction

Austrian out-of-hospital emergency physicians (OOHEP) undergo mandatory biannual emergency physician refresher courses in order to retain their license. The refresher courses are organized by different institutions with the aim to provide education and training in resuscitation and other emergency skills. In addition, ERC-Advanced Life Support (ALS) provider courses and valid recertification refresher courses are offered for emergency physicians with the aim to educate on resuscitation theory, practical skills and attitudes necessary to act safely and efficiently in cardiac arrest situations [1].

In the last decade the approach to treat cardiac arrest has undergone substantial changes with the introduction of mild therapeutic hypothermia. Mild therapeutic hypothermia after cardiac arrest is well known to be associated with favourable outcome and is considered standard treatment [2-4]. Intraosseous access (IO) found its way into the resuscitation guidelines as a substitute to venous access [5]. Strong evidence supports the use of IO in situations when vascular access might be difficult [6,7]. Early defibrillation is the standard treatment for shockable rhythms in patients in cardiac arrest. Therefore, knowing how to use the defibrillator is essential for each physician [8,9]. Currently there is no firm evidence that supports the use of pocket guides in resuscitation, although some providers encourage using them [10].

The present study was part of a larger project evaluating OOHEP in Austria concerning the knowledge about ERC guidelines and how it was implemented in their clinical practice. From that study, we recently published that a higher level of ALS-knowledge was retained if they had participated in an ERC-ALS provider course since 2005 [11].

The aim of the present study was to analyse different reported clinical out-of-hospital emergency medicine skills and knowledge, recommended by the ERC guidelines, and to compare between OOHEP who have taken their recertification and are working regulary and those who are licensed but are currently not or only occasionally working as OOHEP.

## Material and methods

After approval from the Ethics Committee of the Medical University of Vienna (EK Nr: 806/2009) and with written informed consent, the participants of mandatory OOHEP refresher courses were invited to answer a questionnaire including open and multiple choice questions (MCQ) at the beginning of each course over a period of nine months. The results of the questionnaire were correlated first to the OOHEP’s clinical practice and second we checked if the answers were in accordance with the ERC-ALS guidelines. Further, we evaluated if OOHEP who are employed by an ambulance service apply different clinical skills and knowledge compared to OOHEP who are licensed but not currently or only occasionally working as OOHEP (hereinafter the two groups shall be referred to as working vs. inactive OOHEP).

“Working OOHEP” were defined through the following question: “Do you operate as an out-of-hospital emergency physician on a regular basis?”

Four questions assessed the participants’ clinical practice, evaluating knowledge and implementation of the ERC-ALS guidelines. The four questions were:

“Do you initiate mild therapeutic hypothermia after a successful resuscitation?”

“Have you established intraosseous access since 2005?”

“What kind of defibrillator do you use?”

“Do you refer to a pocket guide during resuscitation?”

Additionally, we matched the answers about the study participants’ clinical practice with the MCQ that assessed retention of knowledge (about the recommended course of action for an adult patient with a witnessed cardiac arrest and the defibrillator indicating ventricular fibrillation). The question can be found in Appendix A.

The demographic survey, including age, gender, years of clinical practice, frequency of performed resuscitations and attendance at an ERC-ALS provider course since 2005 was completed. The participants were informed about the survey but had no knowledge of what was assessed. Furthermore, it was ensured that there were no ALS training sessions or lectures at the refresher courses prior to our testing.

### Statistical analysis

Continuous variables are expressed as means ± SD; categorical variables are presented as frequencies and percentages. Differences between continuous variables were tested with Student’s t-test and between dichotomous variables with Fisher’s exact test. Normal distribution for the t-test was given based on the central limit theorem.

The influence of being a working vs. inactive OOHEP on the six dependent variables mentioned above was assessed on the basis of logistic regression models.

We included age (years), gender (1: female, 0: male), duration of clinical experience (years), attending a previous ERC-ALS provider course since 2005 (1: yes, 0: no), and actively providing resuscitation of a patient in cardiac arrest within the last year (1: yes, 0: no) as control variables. All results of the logistic regression model are presented using odds ratios (OR) per unit increase.

A calculation of logistic regression models requires at least 10 events (in the less frequent event category) per predictor variable. Our model was based on six variables, so we needed at least 60 cases in the less frequent category. This held true for our models.

A p value < 0.05 was considered statistically significant. SPSS 16.0 software (SPSS, Chicago, IL) was used for all statistical analyses.

## Results

### Descriptive statistics

The recruitment period lasted nine months. From 19 refresher courses, 81% of the course participants voluntarily took part in this study. Of the 1098 OOHEP who were initially asked to participate, 208 declined. Of the 890 participants who were tested, 54 had to be excluded after testing: 36 because they did not sign the informed consent form and 18 because important data were missing. Therefore, ultimately we analysed data from 836 participants.

The mean age (of the participants) was 40 ± 8 years, and 52% (n = 436) were female. The mean experience in clinical practice was 11 ± 7 years. The comparable demographics of both groups are described in Table 1.

**Table 1:** Descriptive statistics

	**Working OOHEP N = 217**			**Inactive OOHEP N = 619**			
	**Mean ± SD**	**n/%**	**N**	**Mean ± SD**	**n/%**	**N**	**P-2 tailed**
Age [years]	40 ± 8.1		187	40 ± 7.8		516	0.610
Gender: female [number] /%		79/36.4	217		357/57.8	618	< 0.001**
Clinical experience [years]	11.6 ± 7.2		213	11.5 ± 7.1		577	0.850
ERC-ALS Course since 2005		90/42.5	212		230/39.3	585	0.462
Providing a reanimation within the last year		195/90.7	215		236/38.8	608	< 0.001**
Initiated intraosseous access since 2005		41/19.0	216		25/4.1	613	< 0.001**
Referred to a pocket guide during reanimation		27/12.6	214		64/10.9	588	0.529
Initiated mild therapeutic hypothermia		90/42.9	210		84/16.9	498	< 0.001**
Did not know the kind of defibrillator in use		11/6.0	213		173/33.4	518	< 0.001**
Correct answer for MC question about the recommended course of action for an adult patient with a witnessed cardiac arrest and the defibrillator indicating ventricular fibrillation		174/80.2	217		376/60.7	619	< 0.001**

+p < 0,06.

*p < 0,05.

**p < 0,01.

OOHEP: Out-of-hospital emergency physicians.

### Differences between working vs. inactive OOHEP

217 participants (26%) worked regularly as OOHEP and deploy for emergency missions at an average of 15.6 times per month. In the “inactive” group 36 physicians reported that they provide emergency services occasionally with an average of 3.8 emergency missions per month (p < 0.05).

In the group of working OOHEP 36.4% (n = 79) were women, whereas in the other group 57.8% (n = 357) were women.

The percentage of people who have attended an ERC-ALS course since 2005 was about the same in both groups (42.5% vs. 39.3%, p = 0.46). The overall number of physicians that initiated intraosseous access was rather low since 2005 (n = 66, 7.9%) and very few referred to a pocket guide during resuscitation (n = 91, 11.3%).

Bivariate statistics from Table 1 show significant differences between working vs. inactive OOHEP for four out of five dependent variables. The p-values for each of these variables (initiating intraosseous access since 2005, initiating mild therapeutic hypothermia, knowing the kind of defibrillator in use, and correctly answering the question about defibrillation) are below 0.001. Only the use of pocket guides is not significant.

### Results from the logistic regression model

Multivariate logistic regression models are in line with the bivariate findings and are shown in Table 2. Again, the use of pocket guides is the only dependent variable without significant working vs. inactive status. However, the p-value is only slightly above the 0.05 threshold (OR = 1.937, 0.05 < p < 0.06).

**Table 2:** Results from logistic regression analysis

	**Initiated intraosseous access since 2005 (1: yes)**	**Referred to a pocket guide during reanimation (1: yes)**	**Initiated mild therapeutic hypothermia (1: yes)**	**Did not know the kind of defibrillator in use (1: yes)**	**Correct answer to MC question concerning defibrillation (1: yes)**
Working OOHEP (1: yes)	OR: 4.013**	OR: 1.937 +	OR: 2.550**	OR: .203**	OR: 2.292**
	CI: 2.020 - 7.975	CI: .978 - 3.838	CI: 1.589 - 4.090	CI: .097 - .427	CI: 1.434 - 3.665
Age (years)	OR: 1.046	OR: 1.005	OR: .913**	OR: .984	OR: .959*
	CI: .968 - 1.130	CI: .944 - 1.071	CI: .854 - .976	CI: .934 - 1.037	CI: .920 - 1.000
Gender (1: female)	OR: 1.667	OR: 1.225	OR: 1.057	OR: 1.548 +	OR: .807
	CI: .884 - 3.144	CI: .696 - 2.157	CI: .683 - 1.638	CI: .998 - 2.403	CI: .564 - 1.156
Clinical experience (years)	OR: .971	OR: 1.022	OR: 1.086*	OR: 1.005	OR: 1.020
	CI: .891 - 1.058	CI: .955 - 1.095	CI: 1.013 - 1.164	CI: .951 - 1.063	CI: .975 - 1.068
ERC-ALS Course since 2005 (1: yes)	OR: 1.438	OR: 1.140	OR: 1.670*	OR: .697	OR: .809
	CI: .791 - 2.612	CI: .665 - 1.955	CI: 1.103 - 2.528	CI: .452 - 1.075	CI: .571 - 1.147
Provided reanimation within the last year (1: yes)	OR: 2.241 +	OR: .465*	OR: 2.324**	OR: .299**	OR: 1.340
	CI: .979 - 5.129	CI: .245 - .885	CI: 1.389 - 3.889	CI: .190 - .469	CI: .920 - 1.953
N	634	621	551	574	636

+p < 0,06.

*p < 0,05.

**p < 0,01.

Working OOHEP are significantly more likely to have initiated intraosseous access since 2005 (OR = 4.013, p < 0.01). They have also initiated more mild therapeutic hypothermia after successful resuscitation (OR = 2.550, p < 0.01), and their knowledge concerning defibrillation, tested with the MCQ question, was higher (OR = 2.292, p < 0.01). Additionally, inactive OOHEP are less likely to know the kind of defibrillator in use (OR = 0.203, p < 0.01).

Furthermore, physicians who have attended an ERC-ALS provider course since 2005 have initiated more mild therapeutic hypothermia after successful resuscitation (OR = 1.670, p <0.05) as well as participants who resuscitated a victim of cardiac arrest within the last year (OR = 2.324, p < 0.01), while older physicians initiated less hypothermia (OR = 0.913, p <0.01). Gender did not prove to be a significant factor for any of the tested variables.

## Discussion

By analysing OOHEP’s different practice in the field and their implementation of the ERC guidelines in an Austria-wide survey, this study revealed that working OOHEP are more likely to apply current resuscitation standards described in the ERC guidelines, although overall application of the recommendations could be improved considerably.

Advanced cardiac life support knowledge and skills deteriorate rapidly over time [12-16]. Considerable loss of knowledge is evident after six months [14], while skills disappear about one year after the initial training [12,16]. Regular high quality courses and refreshers ought to be mandatory for OOHEP in order to ensure adequate care for critically ill patients. The ERC-ALS provider courses suits this purpose, and participants in these courses show a higher retention of advanced life support knowledge [11]. Additionally, in our study we found that physicians who attended an ERC-ALS provider course are more likely to initiate therapeutic hypothermia after successful ROSC.

Mild therapeutic hypothermia is a well-established treatment after successful resuscitation, especially when there is a short interval between cardiac arrest and ROSC [2-4,17]. Less than 17% of inactive OOHEP said that they initiated mild therapeutic hypothermia after successful ROSC. In contrast, 43% of working OOHEP initiated more than twice the rate of hypothermia. Although this figure is higher, it is still disconcerting, because the initiation of therapeutic hypothermia is an important tool in post-resuscitation care to improve neurologic outcome after surviving cardiac arrest according to the standard guidelines, although recent evidence might lead to changes in this matter [18-20].

These figures are a bit alarming. Even less than half of the ERC-ALS providers did not apply therapeutic hypothermia in spite of the topic being covered extensively during the ERC-ALS course. This means that during the course, instructors and course directors need to point out the importance of this evidence-based intervention, and the ERC might focus more on it in its training program and teaching material.

We also found that OOHEP with more years of clinical experience are more likely to initiate mild therapeutic hypothermia after successful ROSC. This is in contrast to our findings concerning the participants’ age, which is negatively correlated to the initiation of therapeutic hypothermia. That means that age per se is negatively correlated but clinical expertise counts. There is evidence indicating that experience may be linked to a higher level of self-confidence, but not necessarily to improved resuscitation skills [21,22].

Intraosseous access should be established in an emergency situation if peripheral cannot be established within 2 minutes [9]. It is a safe route to deliver a broad variety of drugs as well as fluids [6,7,23-25]. Overall, only 8% of our participants have established intraosseous access since 2005, and the increase to 19% for regularly working OOHEP is significant but still very low. Better and more focused teaching during the courses with available training equipment is needed as well as frequent training rehearsal sessions for this seldom applied practical skill. That is supported by the fact that physicians who frequently perform endotracheal intubation have a higher intubation rate with fewer occurrences of difficult intubations [26]. The cited study argues that emergency physicians should regularly practice these skills, which is best done in a clinical in-hospital setting where the chance of performing the skill is higher.

Simulation has been used as a training method within the field of anaesthesia and emergency medicine, increasing technical as well as non-technical skills and knowledge in a variety of clinical settings [27]. Low and high fidelity simulators seem to be efficient for teaching those procedural skills in a scenario to train the application of therapeutic strategies (like hypothermia) and more technical approaches like IO access. Studies showed that these simulators were superior to conventional didactic teaching in ordinary courses [28-31].

Knowledge about the equipment available and how to apply it is crucial to the proper performance of cardiac life support in an emergency situation. Therefore, we were interested in how well the physicians know “their” type of defibrillator. One third of inactive OOHEP did not know the type of defibrillator in use, whereas only a minority of working OOHEP did not know their equipment. This finding is in accordance with other studies showing that clinically engaged and experienced physicians are more knowledgeable about their resources [12,32].

Less than 11% of our participants refer to a pocket guide during reanimation, with no differences between both groups of OOHEP. There is no evidence to show whether the use of a pocket guide increases the quality of resuscitation. However, one study recommends the use of a pocket guide in preoperative evaluation [10] and recently the use of a set of crisis checklists significantly improved the management of simulated operating-room crises suggesting possible improvement of surgical care [33]. Since we found that the overall adherence to the guidelines is fairly low, the use of pocket guides or checklists during advanced life support might be a suitable way to improve performance with the aim to improve survival after cardiac arrest.

In summary, our study showed that the overall application of current ERC standards is low. Working OOHEP implement the ERC guidelines more effectively into their clinical practice, but the large group of inactive OOHEP with a valid OOHEP license apply important emergency skills at a lower level, according to the recommended European standards. However, the lack of necessary skills and knowledge to properly handle in- or out-of-hospital emergency situations in their day-to-day work may influence the patients' outcome. Consequently, it might be reasonable to call for more teaching and training opportunities, even for working OOHEP, because the application of some important and life-saving rescue techniques (e.g. intraosseous access) and evidence based post resuscitation care strategies (e.g. therapeutic hypothermia) needs to be trained more effectively to increase the rates of application.

### Limitations and strength

There are several limitations to our study. Firstly, we gained the information about the participants’ clinical practice through a questionnaire, while it would have been more accurate to observe the emergency physicians directly in the field. We are aware that linking the tested knowledge and the surveyed practice to the actual real-life emergency performance is limited.

Secondly, our study participants were physicians only, and we obtained only data from Austria. Therefore, our results cannot necessarily be extrapolated to other health professionals or geographic regions.

Thirdly, the evidence level for the induction of mild therapeutic hypothermia is still low and newer evidence might lead to a revision of the current standards in post-resuscitation care.

One strength of this study was the Austria-wide execution of the survey resulting in a high number of participants. We also managed to perform an unannounced survey at the start of the refresher courses with as little chance to prepare for the questions as possible, specifically teaching or training before the courses. We assume that the gathered results reflect what the study participants recalled or are doing in their job on a daily basis.

## Conclusion

Our study showed that the overall application of current ERC standards is low and needs to be trained more effectively. Working OOHEP implement the ERC guidelines better into their clinical practice, but the large group of OOHEP who are licensed but are currently not or only occasionally working apply important emergency skills at a very low level according to European standards.

## Competing interests

This study was supported by a grant from the Austrian Society of Anaesthesiology, Resuscitation and Intensive Care Medicine (ÖGARI). None of the authors has a conflict of interest, but it should be noted that H.F., R.G. teach and direct ERC-ALS courses.

## Authors’ contributions

HF contributed to the concept and design of the study, the acquisition and interpretation of data. He drafted the first version of the manuscript and was responsible for the final version. KB contributed to the data acquisition and statistical analyses. He drafted the manuscript and contributed substantially to the final version of the article. GS as statistician contributed substantially to the statistical analyses and interpretation of data; and revised the article critically until the final version. SN was responsible for the acquisition of data, revised the final article critically. BZ contributed in the acquisition of data and preparation for statistically analysis. CM made substantial contributions to acquisition of data, and revised the article critically. AF contributed to the acquisition of data, the revision of the final article with approval. DS made substantial contributions to acquisition of data and revised the final version of the article critically. RG made substantial contributions to conception and design of the study, the interpretation of data, drafting the article and revising and writing of the final version of the manuscript. All authors read and approved the final manuscript.

## Appendix A

MCQ Question:

What is the recommended course of action for an adult patient with a witnessed cardiac arrest if the defibrillator indicates ventricular fibrillation?

○ Defibrillation once, then check pulse

○ Defibrillation three consecutive times, then check pulse immediately

○ Immediate pulse check, then defibrillation and CPR 30:2

○ No pulse check after 2 min, only CRP 30:2

•Defibrillation, chest compression for 2 min, then rhythm check and pulse check if appropriate
